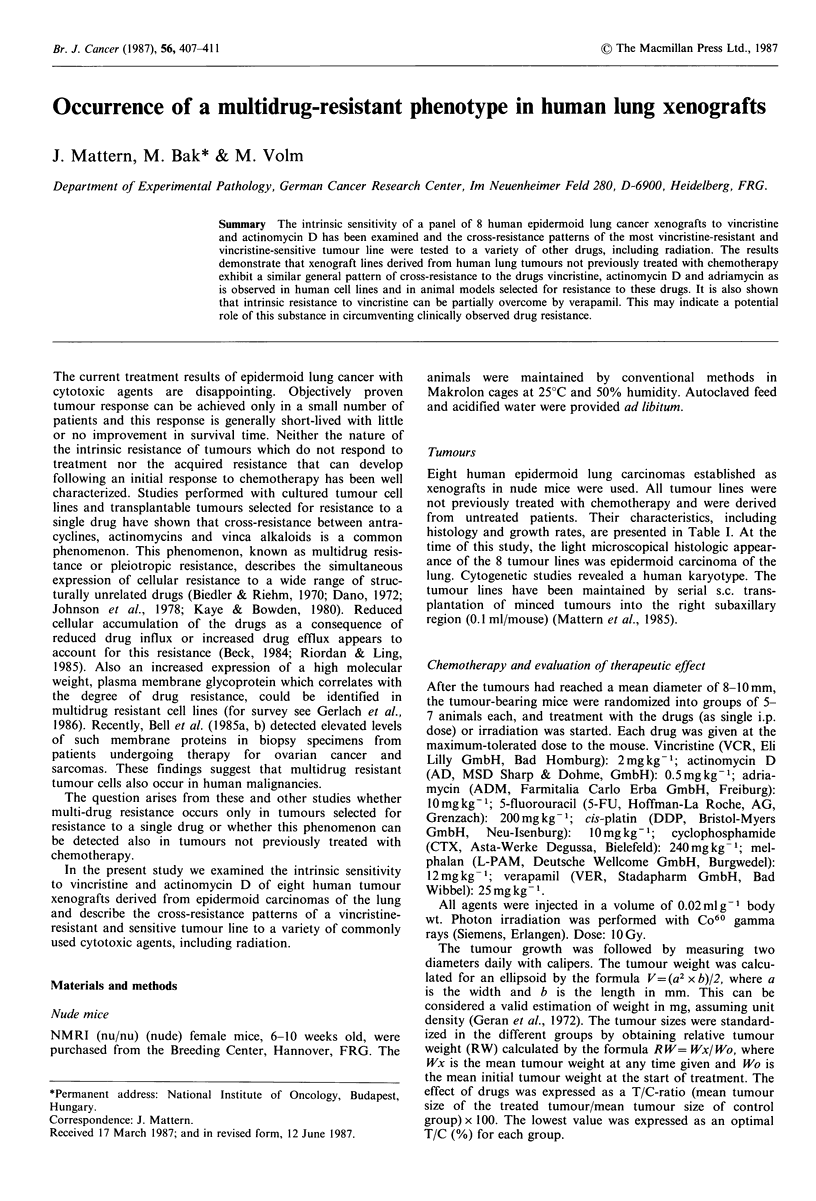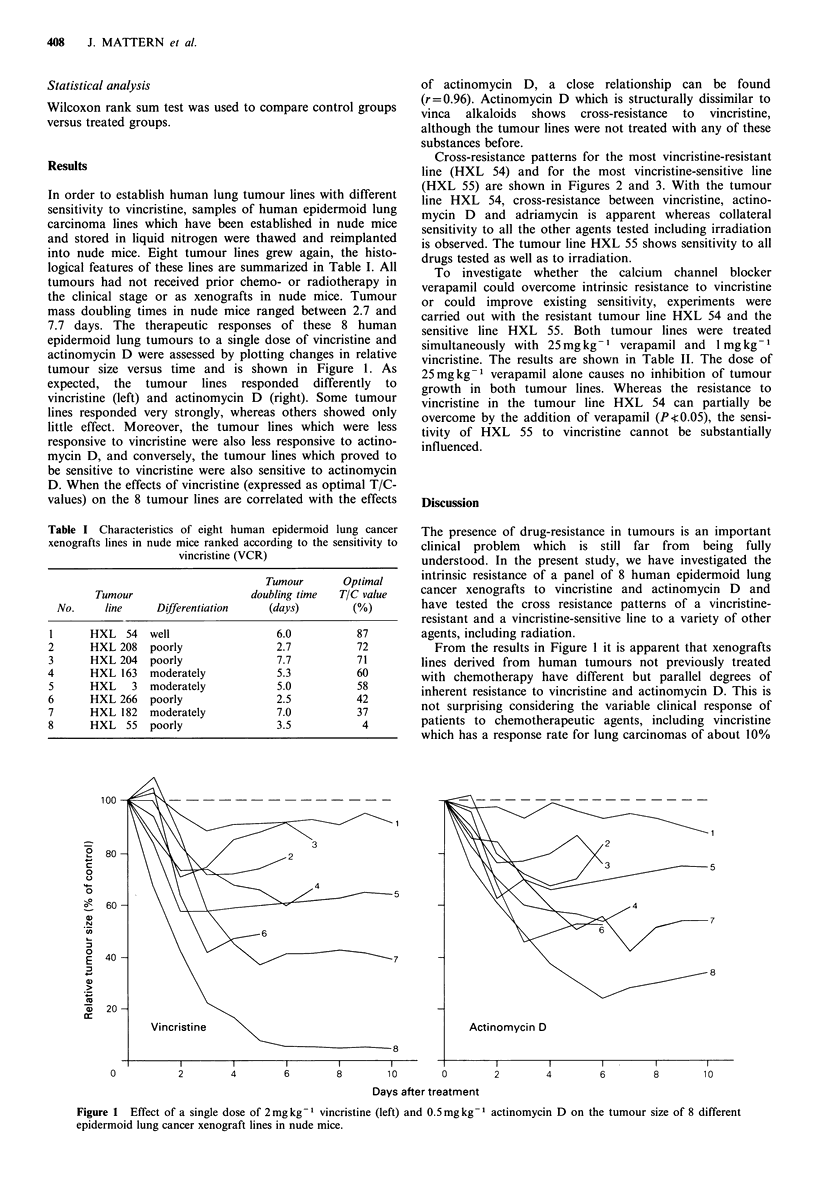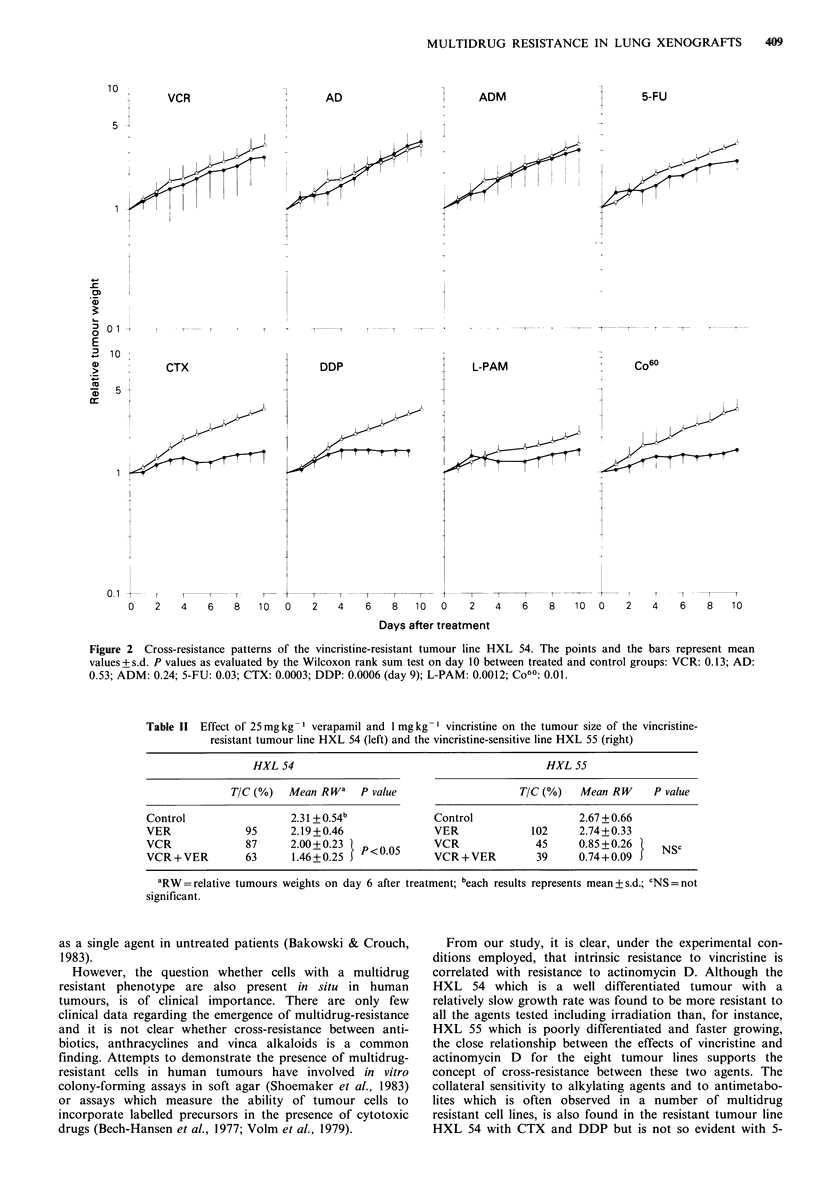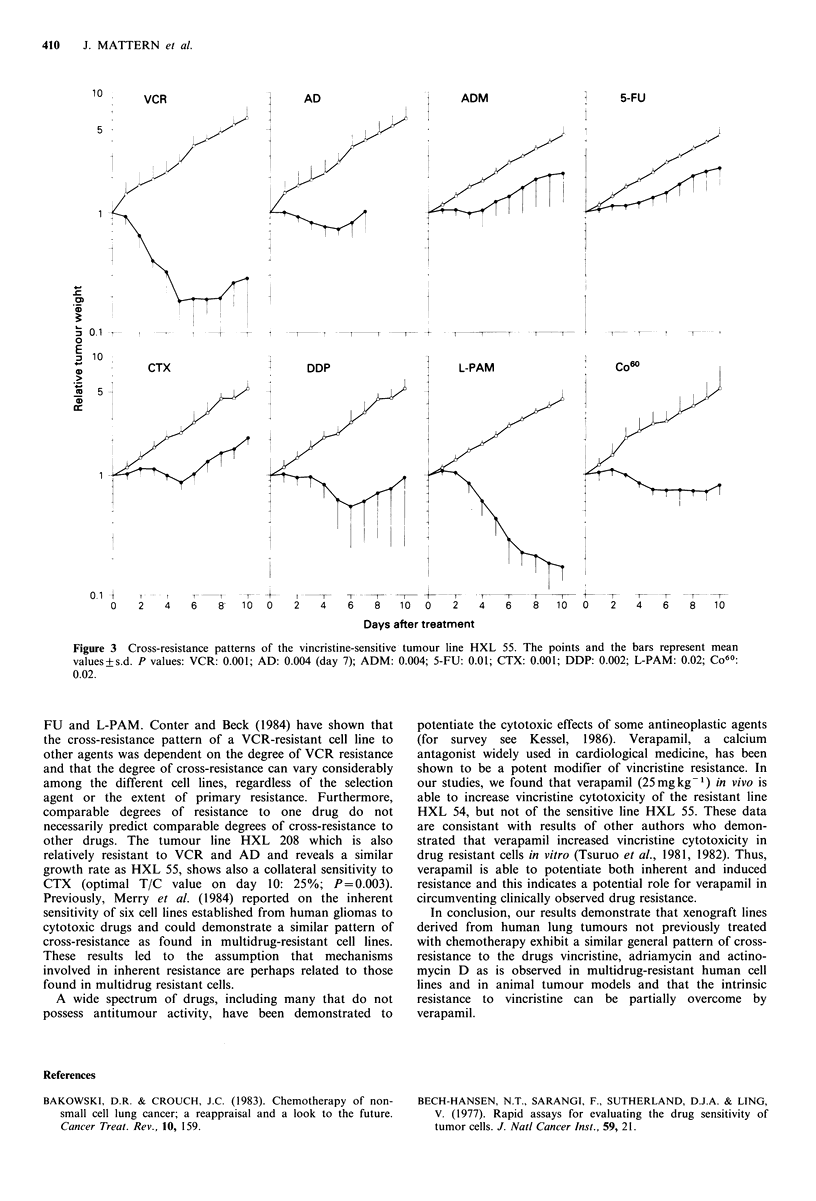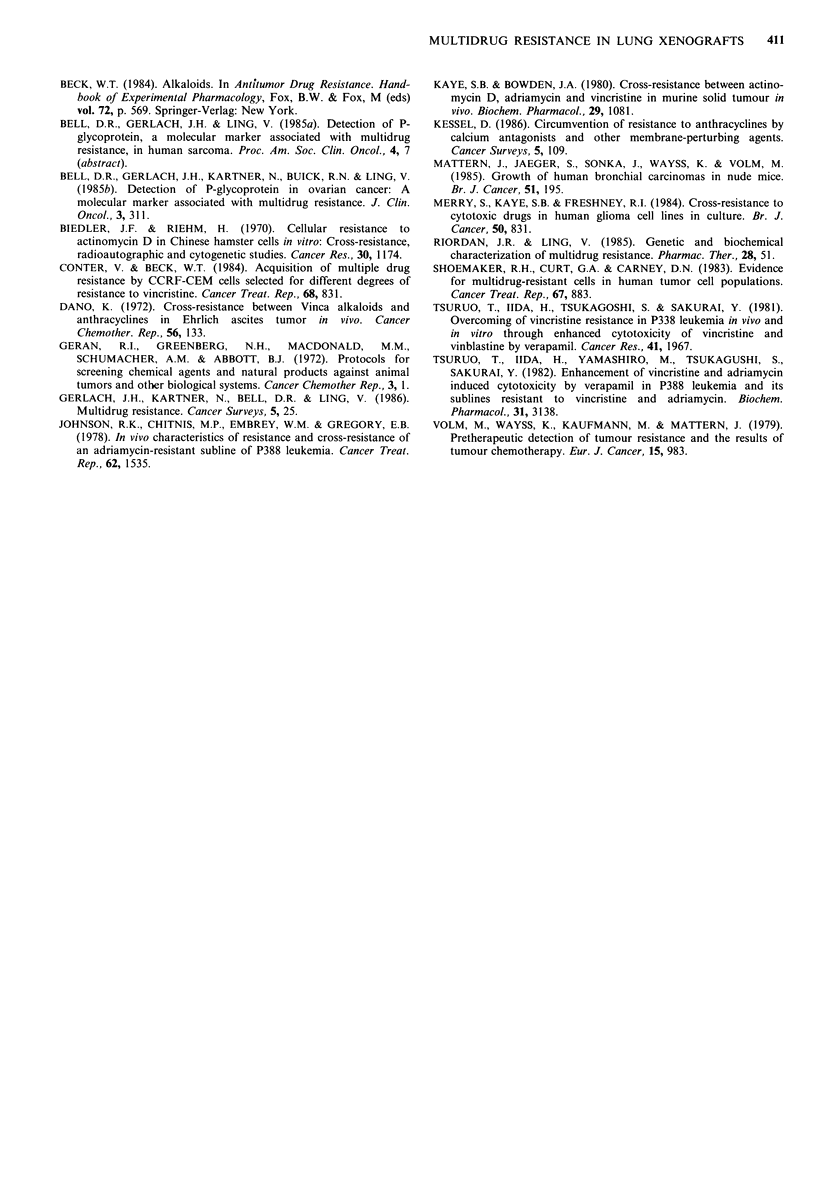# Occurrence of a multidrug-resistant phenotype in human lung xenografts.

**DOI:** 10.1038/bjc.1987.215

**Published:** 1987-10

**Authors:** J. Mattern, M. Bak, M. Volm

**Affiliations:** Department of Experimental Pathology, German Cancer Research Center, Heidelberg.

## Abstract

The intrinsic sensitivity of a panel of 8 human epidermoid lung cancer xenografts to vincristine and actinomycin D has been examined and the cross-resistance patterns of the most vincristine-resistant and vincristine-sensitive tumour line were tested to a variety of other drugs, including radiation. The results demonstrate that xenograft lines derived from human lung tumours not previously treated with chemotherapy exhibit a similar general pattern of cross-resistance to the drugs vincristine, actinomycin D and adriamycin as is observed in human cell lines and in animal models selected for resistance to these drugs. It is also shown that intrinsic resistance to vincristine can be partially overcome by verapamil. This may indicate a potential role of this substance in circumventing clinically observed drug resistance.


*Br. J. Cancer* (1987), **56**, 407–411 © The Macmillan Press Ltd., 1987

### Occurrence of a multidrug-resistant phenotype in human lung xenografts

J. Mattern, M. Bak* & M. Volm

*Department of Experimental Pathology, German Cancer Research Center, Im Neuenheimer Feld 280, D-6900, Heidelberg, FRG.*

**Summary** The intrinsic sensitivity of a panel of 8 human epidermoid lung cancer xenografts to vincristine and actinomycin D has been examined and the cross-resistance patterns of the most vincristine-resistant and vincristine-sensitive tumour line were tested to a variety of other drugs, including radiation. The results demonstrate that xenograft lines derived from human lung tumours not previously treated with chemotherapy exhibit a similar general pattern of cross-resistance to the drugs vincristine, actinomycin D and adriamycin as is observed in human cell lines and in animal models selected for resistance to these drugs. It is also shown that intrinsic resistance to vincristine can be partially overcome by verapamil. This may indicate a potential role of this substance in circumventing clinically observed drug resistance.

The current treatment results of epidermoid lung cancer with cytotoxic agents are disappointing. Objectively proven tumour response can be achieved only in a small number of patients and this response is generally short-lived with little or no improvement in survival time. Neither the nature of the intrinsic resistance of tumours which do not respond to treatment nor the acquired resistance that can develop following an initial response to chemotherapy has been well characterized. Studies performed with cultured tumour cell lines and transplantable tumours selected for resistance to a single drug have shown that cross-resistance between antracyclines, actinomycins and vinca alkaloids is a common phenomenon. This phenomenon, known as multidrug resistance or pleiotropic resistance, describes the simultaneous expression of cellular resistance to a wide range of structurally unrelated drugs (Biedler & Riehm, 1970; Dano, 1972; Johnson *et al.*, 1978; Kaye & Bowden, 1980). Reduced cellular accumulation of the drugs as a consequence of reduced drug influx or increased drug efflux appears to account for this resistance (Beck, 1984; Riordan & Ling, 1985). Also an increased expression of a high molecular weight, plasma membrane glycoprotein which correlates with the degree of drug resistance, could be identified in multidrug resistant cell lines (for survey see Gerlach *et al.*, 1986). Recently, Bell *et al.* (1985a, b) detected elevated levels of such membrane proteins in biopsy specimens from patients undergoing therapy for ovarian cancer and sarcomas. These findings suggest that multidrug resistant tumour cells also occur in human malignancies.

The question arises from these and other studies whether multi-drug resistance occurs only in tumours selected for resistance to a single drug or whether this phenomenon can be detected also in tumours not previously treated with chemotherapy.

In the present study we examined the intrinsic sensitivity to vincristine and actinomycin D of eight human tumour xenografts derived from epidermoid carcinomas of the lung and describe the cross-resistance patterns of a vincristine-resistant and sensitive tumour line to a variety of commonly used cytotoxic agents, including radiation.

### Materials and methods

### *Nude mice*

NMRI (nu/nu) (nude) female mice, 6–10 weeks old, were purchased from the Breeding Center, Hannover, FRG. The animals were maintained by conventional methods in Makrolon cages at 25°C and 50% humidity. Autoclaved feed and acidified water were provided *ad libitum*.

### *Tumours*

Eight human epidermoid lung carcinomas established as xenografts in nude mice were used. All tumour lines were not previously treated with chemotherapy and were derived from untreated patients. Their characteristics, including histology and growth rates, are presented in Table I. At the time of this study, the light microscopical histologic appearance of the 8 tumour lines was epidermoid carcinoma of the lung. Cytogenetic studies revealed a human karyotype. The tumour lines have been maintained by serial s.c. transplantation of minced tumours into the right subaxillary region (0.1 ml/mouse) (Mattern *et al.*, 1985).

### *Chemotherapy and evaluation of therapeutic effect*

After the tumours had reached a mean diameter of 8–10 mm, the tumour-bearing mice were randomized into groups of 5–7 animals each, and treatment with the drugs (as single i.p. dose) or irradiation was started. Each drug was given at the maximum-tolerated dose to the mouse. Vincristine (VCR, Eli Lilly GmbH, Bad Homburg): 2 mg kg$^{-1}$; actinomycin D (AD, MSD Sharp & Dohme, GmbH): 0.5 mg kg$^{-1}$; adriamycin (ADM, Farmitalia Carlo Erba GmbH, Freiburg): 10 mg kg$^{-1}$; 5-fluorouracil (5-FU, Hoffman-La Roche, AG, Grenzach): 200 mg kg$^{-1}$; *cis*-platin (DDP, Bristol-Myers GmbH, Neu-Isenburg): 10 mg kg$^{-1}$; cyclophosphamide (CTX, Asta-Werke Degussa, Bielefeld): 240 mg kg$^{-1}$; melphalan (L-PAM, Deutsche Wellcome GmbH, Burgwedel): 12 mg kg$^{-1}$; verapamil (VER, Stadapharm GmbH, Bad Wibbel): 25 mg kg$^{-1}$.

All agents were injected in a volume of 0.02 ml g$^{-1}$ body wt. Photon irradiation was performed with Co$^{60}$ gamma rays (Siemens, Erlangen). Dose: 10 Gy.

The tumour growth was followed by measuring two diameters daily with calipers. The tumour weight was calculated for an ellipsoid by the formula $V=(a^2 \times b)/2$, where $a$ is the width and $b$ is the length in mm. This can be considered a valid estimation of weight in mg, assuming unit density (Geran *et al.*, 1972). The tumour sizes were standardized in the different groups by obtaining relative tumour weight (RW) calculated by the formula $RW = Wx/Wo$, where $Wx$ is the mean tumour weight at any time given and $Wo$ is the mean initial tumour weight at the start of treatment. The effect of drugs was expressed as a T/C-ratio (mean tumour size of the treated tumour/mean tumour size of control group) × 100. The lowest value was expressed as an optimal T/C (%) for each group.

*Permanent address: National Institute of Oncology, Budapest, Hungary.
Correspondence: J. Mattern.
Received 17 March 1987; and in revised form, 12 June 1987.

*Statistical analysis*

Wilcoxon rank sum test was used to compare control groups versus treated groups.

### Results

In order to establish human lung tumour lines with different sensitivity to vincristine, samples of human epidermoid lung carcinoma lines which have been established in nude mice and stored in liquid nitrogen were thawed and reimplanted into nude mice. Eight tumour lines grew again, the histological features of these lines are summarized in Table I. All tumours had not received prior chemo- or radiotherapy in the clinical stage or as xenografts in nude mice. Tumour mass doubling times in nude mice ranged between 2.7 and 7.7 days. The therapeutic responses of these 8 human epidermoid lung tumours to a single dose of vincristine and actinomycin D were assessed by plotting changes in relative tumour size versus time and is shown in Figure 1. As expected, the tumour lines responded differently to vincristine (left) and actinomycin D (right). Some tumour lines responded very strongly, whereas others showed only little effect. Moreover, the tumour lines which were less responsive to vincristine were also less responsive to actinomycin D, and conversely, the tumour lines which proved to be sensitive to vincristine were also sensitive to actinomycin D. When the effects of vincristine (expressed as optimal T/C-values) on the 8 tumour lines are correlated with the effects of actinomycin D, a close relationship can be found ($r = 0.96$). Actinomycin D which is structurally dissimilar to vinca alkaloids shows cross-resistance to vincristine, although the tumour lines were not treated with any of these substances before.

**Table I** Characteristics of eight human epidermoid lung cancer xenografts lines in nude mice ranked according to the sensitivity to vincristine (VCR)

| *No.* | *Tumour line* | *Differentiation* | *Tumour doubling time (days)* | *Optimal T/C value (%)* |
|---|---|---|---|---|
| 1 | HXL 54 | well | 6.0 | 87 |
| 2 | HXL 208 | poorly | 2.7 | 72 |
| 3 | HXL 204 | poorly | 7.7 | 71 |
| 4 | HXL 163 | moderately | 5.3 | 60 |
| 5 | HXL 3 | moderately | 5.0 | 58 |
| 6 | HXL 266 | poorly | 2.5 | 42 |
| 7 | HXL 182 | moderately | 7.0 | 37 |
| 8 | HXL 55 | poorly | 3.5 | 4 |

Cross-resistance patterns for the most vincristine-resistant line (HXL 54) and for the most vincristine-sensitive line (HXL 55) are shown in Figures 2 and 3. With the tumour line HXL 54, cross-resistance between vincristine, actinomycin D and adriamycin is apparent whereas collateral sensitivity to all the other agents tested including irradiation is observed. The tumour line HXL 55 shows sensitivity to all drugs tested as well as to irradiation.

To investigate whether the calcium channel blocker verapamil could overcome intrinsic resistance to vincristine or could improve existing sensitivity, experiments were carried out with the resistant tumour line HXL 54 and the sensitive line HXL 55. Both tumour lines were treated simultaneously with $25\,\mathrm{mg\,kg^{-1}}$ verapamil and $1\,\mathrm{mg\,kg^{-1}}$ vincristine. The results are shown in Table II. The dose of $25\,\mathrm{mg\,kg^{-1}}$ verapamil alone causes no inhibition of tumour growth in both tumour lines. Whereas the resistance to vincristine in the tumour line HXL 54 can partially be overcome by the addition of verapamil ($P \nless 0.05$), the sensitivity of HXL 55 to vincristine cannot be substantially influenced.

### Discussion

The presence of drug-resistance in tumours is an important clinical problem which is still far from being fully understood. In the present study, we have investigated the intrinsic resistance of a panel of 8 human epidermoid lung cancer xenografts to vincristine and actinomycin D and have tested the cross resistance patterns of a vincristine-resistant and a vincristine-sensitive line to a variety of other agents, including radiation.

From the results in Figure 1 it is apparent that xenografts lines derived from human tumours not previously treated with chemotherapy have different but parallel degrees of inherent resistance to vincristine and actinomycin D. This is not surprising considering the variable clinical response of patients to chemotherapeutic agents, including vincristine which has a response rate for lung carcinomas of about 10%

**Figure 1** Effect of a single dose of $2\,\mathrm{mg\,kg^{-1}}$ vincristine (left) and $0.5\,\mathrm{mg\,kg^{-1}}$ actinomycin D on the tumour size of 8 different epidermoid lung cancer xenograft lines in nude mice.

**Figure 2** Cross-resistance patterns of the vincristine-resistant tumour line HXL 54. The points and the bars represent mean values ± s.d. $P$ values as evaluated by the Wilcoxon rank sum test on day 10 between treated and control groups: VCR: 0.13; AD: 0.53; ADM: 0.24; 5-FU: 0.03; CTX: 0.0003; DDP: 0.0006 (day 9); L-PAM: 0.0012; $Co^{60}$: 0.01.

**Table II** Effect of $25\,mg\,kg^{-1}$ verapamil and $1\,mg\,kg^{-1}$ vincristine on the tumour size of the vincristine-resistant tumour line HXL 54 (left) and the vincristine-sensitive line HXL 55 (right)

| | *HXL 54* | | | | *HXL 55* | | |
|---|---|---|---|---|---|---|---|
| | *T/C* (%) | *Mean RW*[a] | *P value* | | *T/C* (%) | *Mean RW* | *P value* |
| Control | | 2.31 ± 0.54[b] | | Control | | 2.67 ± 0.66 | |
| VER | 95 | 2.19 ± 0.46 | | VER | 102 | 2.74 ± 0.33 | |
| VCR | 87 | 2.00 ± 0.23 | $P<0.05$ | VCR | 45 | 0.85 ± 0.26 | NS[c] |
| VCR + VER | 63 | 1.46 ± 0.25 | | VCR + VER | 39 | 0.74 + 0.09 | |

[a]RW = relative tumours weights on day 6 after treatment; [b]each results represents mean ± s.d.; [c]NS = not significant.

as a single agent in untreated patients (Bakowski & Crouch, 1983).

However, the question whether cells with a multidrug resistant phenotype are also present *in situ* in human tumours, is of clinical importance. There are only few clinical data regarding the emergence of multidrug-resistance and it is not clear whether cross-resistance between antibiotics, anthracyclines and vinca alkaloids is a common finding. Attempts to demonstrate the presence of multidrug-resistant cells in human tumours have involved *in vitro* colony-forming assays in soft agar (Shoemaker *et al.*, 1983) or assays which measure the ability of tumour cells to incorporate labelled precursors in the presence of cytotoxic drugs (Bech-Hansen *et al.*, 1977; Volm *et al.*, 1979).

From our study, it is clear, under the experimental conditions employed, that intrinsic resistance to vincristine is correlated with resistance to actinomycin D. Although the HXL 54 which is a well differentiated tumour with a relatively slow growth rate was found to be more resistant to all the agents tested including irradiation than, for instance, HXL 55 which is poorly differentiated and faster growing, the close relationship between the effects of vincristine and actinomycin D for the eight tumour lines supports the concept of cross-resistance between these two agents. The collateral sensitivity to alkylating agents and to antimetabolites which is often observed in a number of multidrug resistant cell lines, is also found in the resistant tumour line HXL 54 with CTX and DDP but is not so evident with 5-

**Figure 3** Cross-resistance patterns of the vincristine-sensitive tumour line HXL 55. The points and the bars represent mean values ± s.d. *P* values: VCR: 0.001; AD: 0.004 (day 7); ADM: 0.004; 5-FU: 0.01; CTX: 0.001; DDP: 0.002; L-PAM: 0.02; $Co^{60}$: 0.02.

FU and L-PAM. Conter and Beck (1984) have shown that the cross-resistance pattern of a VCR-resistant cell line to other agents was dependent on the degree of VCR resistance and that the degree of cross-resistance can vary considerably among the different cell lines, regardless of the selection agent or the extent of primary resistance. Furthermore, comparable degrees of resistance to one drug do not necessarily predict comparable degrees of cross-resistance to other drugs. The tumour line HXL 208 which is also relatively resistant to VCR and AD and reveals a similar growth rate as HXL 55, shows also a collateral sensitivity to CTX (optimal T/C value on day 10: 25%; $P = 0.003$). Previously, Merry *et al.* (1984) reported on the inherent sensitivity of six cell lines established from human gliomas to cytotoxic drugs and could demonstrate a similar pattern of cross-resistance as found in multidrug-resistant cell lines. These results led to the assumption that mechanisms involved in inherent resistance are perhaps related to those found in multidrug resistant cells.

A wide spectrum of drugs, including many that do not possess antitumour activity, have been demonstrated to potentiate the cytotoxic effects of some antineoplastic agents (for survey see Kessel, 1986). Verapamil, a calcium antagonist widely used in cardiological medicine, has been shown to be a potent modifier of vincristine resistance. In our studies, we found that verapamil (25 mg kg$^{-1}$) *in vivo* is able to increase vincristine cytotoxicity of the resistant line HXL 54, but not of the sensitive line HXL 55. These data are consistant with results of other authors who demonstrated that verapamil increased vincristine cytotoxicity in drug resistant cells *in vitro* (Tsuruo *et al.*, 1981, 1982). Thus, verapamil is able to potentiate both inherent and induced resistance and this indicates a potential role for verapamil in circumventing clinically observed drug resistance.

In conclusion, our results demonstrate that xenograft lines derived from human lung tumours not previously treated with chemotherapy exhibit a similar general pattern of cross-resistance to the drugs vincristine, adriamycin and actinomycin D as is observed in multidrug-resistant human cell lines and in animal tumour models and that the intrinsic resistance to vincristine can be partially overcome by verapamil.

### References

BAKOWSKI, D.R. & CROUCH, J.C. (1983). Chemotherapy of non-small cell lung cancer; a reappraisal and a look to the future. *Cancer Treat. Rev.*, **10**, 159.

BECH-HANSEN, N.T., SARANGI, F., SUTHERLAND, D.J.A. & LING, V. (1977). Rapid assays for evaluating the drug sensitivity of tumor cells. *J. Natl Cancer Inst.*, **59**, 21.

BECK, W.T. (1984). Alkaloids. In *Antitumor Drug Resistance. Handbook of Experimental Pharmacology*, Fox, B.W. & Fox, M (eds) **vol. 72,** p. 569. Springer-Verlag: New York.

BELL, D.R., GERLACH, J.H. & LING, V. (1985*a*). Detection of P-glycoprotein, a molecular marker associated with multidrug resistance, in human sarcoma. *Proc. Am. Soc. Clin. Oncol.,* **4,** 7 (*abstract*).

BELL, D.R., GERLACH, J.H., KARTNER, N., BUICK, R.N. & LING, V. (1985*b*). Detection of P-glycoprotein in ovarian cancer: A molecular marker associated with multidrug resistance. *J. Clin. Oncol.,* **3,** 311.

BIEDLER, J.F. & RIEHM, H. (1970). Cellular resistance to actinomycin D in Chinese hamster cells *in vitro*: Cross-resistance, radioautographic and cytogenetic studies. *Cancer Res.,* **30,** 1174.

CONTER, V. & BECK, W.T. (1984). Acquisition of multiple drug resistance by CCRF-CEM cells selected for different degrees of resistance to vincristine. *Cancer Treat. Rep.,* **68,** 831.

DANO, K. (1972). Cross-resistance between Vinca alkaloids and anthracyclines in Ehrlich ascites tumor *in vivo. Cancer Chemother. Rep.,* **56,** 133.

GERAN, R.I., GREENBERG, N.H., MACDONALD, M.M., SCHUMACHER, A.M. & ABBOTT, B.J. (1972). Protocols for screening chemical agents and natural products against animal tumors and other biological systems. *Cancer Chemother Rep.,* **3,** 1.

GERLACH, J.H., KARTNER, N., BELL, D.R. & LING, V. (1986). Multidrug resistance. *Cancer Surveys,* **5,** 25.

JOHNSON, R.K., CHITNIS, M.P., EMBREY, W.M. & GREGORY, E.B. (1978). *In vivo* characteristics of resistance and cross-resistance of an adriamycin-resistant subline of P388 leukemia. *Cancer Treat. Rep.,* **62,** 1535.

KAYE, S.B. & BOWDEN, J.A. (1980). Cross-resistance between actinomycin D, adriamycin and vincristine in murine solid tumour *in vivo. Biochem. Pharmacol.,* **29,** 1081.

KESSEL, D. (1986). Circumvention of resistance to anthracyclines by calcium antagonists and other membrane-perturbing agents. *Cancer Surveys,* **5,** 109.

MATTERN, J., JAEGER, S., SONKA, J., WAYSS, K. & VOLM, M. (1985). Growth of human bronchial carcinomas in nude mice. *Br. J. Cancer,* **51,** 195.

MERRY, S., KAYE, S.B. & FRESHNEY, R.I. (1984). Cross-resistance to cytotoxic drugs in human glioma cell lines in culture. *Br. J. Cancer,* **50,** 831.

RIORDAN, J.R. & LING, V. (1985). Genetic and biochemical characterization of multidrug resistance. *Pharmac. Ther.,* **28,** 51.

SHOEMAKER, R.H., CURT, G.A. & CARNEY, D.N. (1983). Evidence for multidrug-resistant cells in human tumor cell populations. *Cancer Treat. Rep.,* **67,** 883.

TSURUO, T., IIDA, H., TSUKAGOSHI, S. & SAKURAI, Y. (1981). Overcoming of vincristine resistance in P338 leukemia *in vivo* and *in vitro* through enhanced cytotoxicity of vincristine and vinblastine by verapamil. *Cancer Res.,* **41,** 1967.

TSURUO, T., IIDA, H., YAMASHIRO, M., TSUKAGUSHI, S., SAKURAI, Y. (1982). Enhancement of vincristine and adriamycin induced cytotoxicity by verapamil in P388 leukemia and its sublines resistant to vincristine and adriamycin. *Biochem. Pharmacol.,* **31,** 3138.

VOLM, M., WAYSS, K., KAUFMANN, M. & MATTERN, J. (1979). Pretherapeutic detection of tumour resistance and the results of tumour chemotherapy. *Eur. J. Cancer,* **15,** 983.